# The influence of COVID-19 pandemic on body mass and cardiopulmonary endurance of Chinese adolescents: a longitudinal follow-up study

**DOI:** 10.3389/fpubh.2024.1406120

**Published:** 2024-08-07

**Authors:** Hao Cheng, Long Jiao, Bin Liu

**Affiliations:** ^1^College of Physical Education, Shandong Sport University, Jinan, Shandong, China; ^2^Basic Education Group, Shandong Normal University, Jinan, Shandong, China; ^3^Physical Education Teaching and Research Group, Jinan Licheng No.2 High School, Jinan, Shandong, China

**Keywords:** body mass index, COVID-19, cardiopulmonary endurance, adolescents, follow-up study

## Abstract

**Background:**

With the spread and spread of COVID-19 around the world, youth’s learning, lifestyle and health have been greatly affected. Based on the current research, there is no adequate analysis of the development of young people’s physique and heart and lung health during COVID-19, and there is a lack of relevant targeted research. The aim of this study was to investigate the changes of BMI and Maximum Oxygen Absorption (VO_2max_) in 12–14 year old teenagers before and after COVID-19.

**Method:**

The BMI, 1,000/800 m running time and associated data related to 29,813 individuals between 2019 and 2022 were collected by cluster sampling, and the changes of BMI Z and VO_2max_ before and after the outbreak were analyzed. Moreover, the relationship between BMI and cardiovascular endurance was analyzed by means of multi-linear stepwise regression.

**Results:**

The covariance analysis models indicated that compared with 2019, adolescent weight, BMI, and 1,000/800 m running time showed varying degrees of growth in 2020, while lung capacity decreased. All indicators achieved rapid rebound in 2021 and 2022 (*p* < 0.01); the one-way analysis of variance models indicated that The BMI Z score and VO_2max_ of adolescents showed growth and decline in 2020, respectively, and achieved rapid recovery and development in 2021 and 2022 (*p* < 0.01). The results of the multiple linear stepwise regression analysis indicate that, after the years of BMI Z and novel coronavirus infection were included (△*R^2^* = 0.179), adolescents’ overweight and obesity were positively correlated with the maximum oxygen uptake (*B* = 0.643, 95%*CI* = 0.634 ~ 0.652); There is a negative correlation between weight loss and maximum oxygen uptake (*B* = −0.510, 95%*CI* = −0.537~−0.484); The year of novel coronavirus infection was positively correlated with the maximum oxygen uptake of adolescents (*B* = 0.116, 95%*CI* = 0.107~0.125).

**Conclusion:**

This study shows that the impact of COVID-19 on BMI and heart and lung health in adolescents is significant. Young people of all ages and sexes showed similar developmental trends.

## Highlights

- Adolescent weight, BMI, and 1,000/800 m running time in 2020 have increased to varying degrees, but lung capacity has declined. All indicators were controlled and restored in 2021 and 2022.
- There was a positive correlation between the year of new coronavirus infection and the maximal oxygen absorption.

## Introduction

Corona virus (COVID-19) appeared and spread in China in late 2019. As the epidemic spreads, scientists expect a major global health event in 2020 (1). The characteristics of COVID-19 are uncertain, difficult to prevent and control, and their rapid spread (2), posing a fundamental threat to the physical and psychological health of individuals and to the normal development of the social economy (3). To control the spread of new coronavirus, the Chinese Ministry of Education issued the “Overall Planning for Preventing and Controlling COVID-19 in the Education System” and “Reforming and Developing Educational Systems,” which delayed the opening time of offline classes, and started on-line learning (4). Prolonged home protection, extended screen time, reduced time spent outdoors, and longer sitting hours could also result in negative consequences, including weight gain and reduced cardiovascular health in adolescents (5). In 2020, when the COVID-19 outbreak broke out, the percentage of adolescents who are overweight and obese, as well as their average body weight, showed an upward trend (6). Therefore, it is essential to maintain optimal health, weight and heart rate monitoring in adolescents.

During adolescence, a crucial time for children to become adults, they develop physically and mentally (7). Young people’s self-consciousness increases as they get older, their emotional stability gets worse, and their body and mind are more susceptible to COVID-19 transmission (8). In the period of COVID-19, the decrease of outdoor activities and the increasing amount of sedentary time in the family had great influence on the body shape, diet habits, chronic disease management, heart and lung health and exercise habits. During the period of COVID-19, some surveys observed an upward trend in the consumption of weight, carbohydrates and drinks among adolescents (9–11). The emergence of the COVID-19 has also brought difficulties to the management and treatment of chronic diseases such as asthma, allergies and allergic rhinitis in children (12, 13). Moreover, it was found that the epidemic had a significant effect on aerobic activity time and outdoor activity ratio (14, 15). Research also shows that the COVID-19 epidemic has long-term sequelae for adolescents, and the most prevalent symptom is fatigue (16). However, the present study on the development trend of the body shape and heart function of young people in China before and after COVID-19 is not clear, and there is a lack of relevant targeted research.

The purpose of this research was to analyze the changes of body shape and heart and lung health of youth in China before and after the outbreak of COVID-19. Our hypothesis is that the prevalence of COVID-19 has a significant impact on young people’s physical fitness and cardiac endurance.

## Materials and methods

### Subjects

The data were collected from the National Physical Fitness Survey in Jinan, Shandong Province, from 2019 to 2022. 12–14-year-old children completed the physical fitness tests. Both participants and their parents (or guardians) gave their informed consent for all participants. The study protocol was approved by Shandong Normal University Ethics Committee (2021066).

### Procedures

We performed anthropometric measurements in accordance with technical standards, including the National Student Physical Health Standard. Intelligent physical health monitoring devices were used, for example, GMCS-SGJ3 and Weight Testing Instrument (GMCS-RCS3), and we used non-contact measurement with multi-point infrared sensor array to record the student’s data automatically and store them in the system.

#### Height and weight

During the measurement of young people’s height and weight, the students should remove their shoes and caps, and stand on the base of the instrument with their back to the column. The upper limbs are naturally drooping, and the legs are straight. Students are required to hold their heels together, their toes are about 60 degrees away from each other, and their heels, sacrum, and shoulder blades are in contact with the column. Precision shall be set to one decimal point (17). Young people’s height and weight have only been measured once.

### BMI calculation

The BMI is computed as follows: BMI = body mass (kg)/height (m)^2^. According to the World Health Organization (WHO) criteria, the students were classified into four groups: < 18.5 kg/m^2^, 18.5~23.9 kg/m^2^, 24–27.9 kg/m^2^, and ≥ 28 kg/m^2^, respectively (18). Based on the WHO growth curve (19) for children and adolescents (19), each participant’s BMI Z score was calculated as follows: BMI Z Score = (Measure Value − Gender Benchmark Mean)/Standard Deviation (SSRS) (20). A BMI Z of >1 is indicative of overweight in adolescents; a BMI of over 2 indicates obesity in adolescents, and a BMI of < −2 is indicative of adolescent weight loss (21).

#### 800/1,000 m run

Every student stands at the beginning of the race and is required to finish the 800 or 1,000 meters as quickly as possible. Minutes and seconds were recorded. Female students ran 800 m run, while male students ran 1,000 m.

### VO_2max_ calculation

The maximal oxygen uptake (VO_2max_) of adolescents was calculated on the basis of their performance at 1,000/800 m run, using a calculation formula developed by Liu Haiyun on the basis of the Chinese 1,000/800 m Youth Cardiovascular Endurance Test Project (22): VO_2max_ (L/min) = 1.640–0.004 x gender (1 male, 2 females) × time (s) + 0.037 × body weight (kg).

### Calculation

#### Statistical analysis

The IBM SPSS statistical software (version 26.0, Chicago, IL, United States) was used to process experimental data. All data were presented as “mean ± standard deviation” (M ± SD). Using the age-adjusted covariate analysis model, the basic indexes of male and female students before and after SARS were compared. The BMI and the maximal oxygen uptake of high school students were compared with those of different ages and sexes. The relationship between BMI and cardiopulmonary endurance in junior high school students was investigated by means of multi-linear stepwise regression. Model 1 included the age and sex of middle school students, and model 2 included BMI Z score and new coronavirus infection year, = 0.05, and the significance of all tests was *p* < 0.05.

## Results

### Descriptive analysis

In this study, 93,046 teenagers aged 12–14 years in Jinan City during 2019~2022 were investigated. From 2019 to 2022, 7,333, 7,574, 7,616, 7,290 individuals were collected, of which 15,626 (52.41%) and 14,187 (47.59%). There were no statistical differences in the age of boys and girls between 2019 and 2022 (*p* > 0.05). Covariates analysis indicates that the total height of adolescents in 2019~2022 presents a continuous increasing tendency. In 2020, measures like weight, BMI, and 1,000/800 m running time of adolescents showed varying degrees of growth in comparison with 2019, while lung capacity declined. All indicators achieved rapid recovery and rebound in 2021 and 2022. The changes of height, weight, BMI, vitality and 1,000/800 m running performance of young people were statistically significant (*p* < 0.01; Table 1).

**Table 1:** Mean and standard deviation of age, height, weight, BMI, vital capacity, 800/1,000 m run of Adolescents in Jinan City from the years 2019 to 2022.

Index	Gender	2019	2020	2021	2022	*F*	*P*
Age	Male	12.98 ± 0.83	12.98 ± 0.80	13.02 ± 0.82	13.00 ± 0.82	3.53	0.09
	Female	12.97 ± 0.83	12.99 ± 0.81	13.02 ± 0.81	13.01 ± 0.83	3.21	0.06
Height (cm)	Male	167.81 ± 8.58	168.00 ± 9.06	169.05 ± 8.33	169.10 ± 8.83	23.61	<0.01
	Female	161.33 ± 5.96	161.68 ± 6.05	161.90 ± 5.89	162.06 ± 5.98	9.79	<0.01
Weight (kg)	Male	62.33 ± 15.74	64.96 ± 16.77	64.41 ± 17.10	64.19 ± 16.43	18.89	<0.01
	Female	53.99 ± 11.21	55.94 ± 12.32	55.57 ± 12.29	55.19 ± 12.42	16.95	<0.01
BMI (kg·m^−2^)	Male	21.94 ± 4.76	22.70 ± 5.04	22.57 ± 4.88	22.29 ± 4.79	18.58	<0.01
	Female	20.67 ± 3.77	21.27 ± 4.16	21.08 ± 4.11	21.04 ± 4.18	13.05	<0.01
Vital capacity (mL)	Male	3505.46 ± 817.39	3288.05 ± 914.17	3723.94 ± 1015.82	3641.82 ± 898.08	170.64	<0.01
	Female	2831.66 ± 602.37	2635.85 ± 737.55	2909.68 ± 754.50	2884.92 ± 672.88	114.28	<0.01
800/1,000 m run (m/s)	Male	291.52 ± 57.99	303.78 ± 57.86	275.22 ± 49.58	274.61 ± 51.76	259.19	<0.01
	Female	252.22 ± 39.51	256.78 ± 35.85	241.25 ± 31.41	241.51 ± 31.75	176.33	<0.01

In 2020, the BMI of 12–14 year olds in Jinan City was significantly higher than in 2019, and was higher than the highest in 2019, 2021 and 2022 (2019: 0.48 ± 0.74; 2020: 0.58 ± 0.80; 2021: 0.57 ± 0.77; 2022: 0.53 ± 0.76; *F* = 22.65, *p* < 0.01). The BMI Z scores of 6–14 year olds in all age groups showed similar trends, with BMI Z rising significantly in 2020 and declining in 2021 and 2022. Moreover, the VO2 max of young people from 12 to 14 years old in Jinan City decreased from 2019 to 2019, and was below the minimum in 2019, 2021 and 2022 (2019: 2.25 ± 0.81; 2020: 2.24 ± 0.79; 2021: 2.40 ± 0.78; 2022: 2.37 ± 0.79; *F* = 78.80, *p* < 0.01). The VO_2max_ in children 6 to 14 years old shows a similar trend in all age groups, with VO_2MAX_ falling in 2020 and rising in 2021 and 2022.

### Regressive analysis

With the maximum oxygen uptake as the dependent variable, model 1 included age (continuous variable), gender (male = 1, female = 2), model 2 included BMI Z score (normal weight = 1, overweight and obesity = 2, emaciation = 3) and novel coronavirus infection years (2019, 2020 = 1; 2021, 2022 = 2), and then conducted multiple linear stepwise regression analysis. Table 2 shows that after the model 2 includes the BMI Z score and the year of novel coronavirus infection (△*R^2^* = 0.179), it shows that 17.9% of the changes in cardiopulmonary endurance of middle school students can be explained by the BMI Z score and the year of novel coronavirus infection (*p* < 0.01). Compared with adolescents with normal body size, the degree of overweight and obesity in adolescents (B = 0.643, 95% CI = 0.634~0.652) is positively correlated with maximum oxygen uptake (*p* < 0.01); The degree of low weight in adolescents (*B* =−0.510, 95%*CI* = −0.537 ~ −0.484) is negatively correlated with maximum oxygen uptake (*p* < 0.01); The year of novel coronavirus infection (*B* = 0.116, 95%*CI* = 0.107~0.125) was positively correlated with the maximum oxygen uptake of adolescents (*p* < 0.01).

**Table 2:** Mean and standard deviation of BMI Z score and VO_2max_ of Adolescents in Jinan City from the years 2019 to 2022.

Gender	Age	Index	2019	2020	2021	2022	*F*	*P*
Male	12	BMI Z	0.77 ± 0.87	0.90 ± 0.90	0.85 ± 0.86	0.82 ± 0.87	4.85	<0.01
		VO_2max_	2.53 ± 0.54	2.49 ± 0.54	2.67 ± 0.55	2.58 ± 0.56	29.88	<0.01
	13	BMI Z	0.63 ± 0.80	0.78 ± 0.86	0.74 ± 0.87	0.67 ± 0.80	8.20	<0.01
		VO_2max_	2.76 ± 0.60	2.75 ± 0.56	2.98 ± 0.56	2.95 ± 0.56	56.24	<0.01
	14	BMI Z	0.53 ± 0.78	0.68 ± 0.83	0.62 ± 0.79	0.61 ± 0.79	7.96	<0.01
		VO_2max_	3.15 ± 0.58	3.10 ± 0.50	3.17 ± 0.55	3.18 ± 0.55	5.28	<0.01
Female	12	BMI Z	0.35 ± 0.63	0.43 ± 0.68	0.42 ± 0.64	0.41 ± 0.71	4.51	<0.01
		VO_2max_	1.44 ± 0.46	1.39 ± 0.46	1.62 ± 0.42	1.59 ± 0.47	65.08	<0.01
	13	BMI Z	0.27 ± 0.59	0.38 ± 0.66	0.36 ± 0.63	0.34 ± 0.63	6.41	<0.01
		VO_2max_	1.64 ± 0.42	1.61 ± 0.46	1.78 ± 0.42	1.79 ± 0.42	53.34	<0.01
	14	BMI Z	0.25 ± 0.51	0.32 ± 0.58	0.28 ± 0.61	0.28 ± 0.58	4.19	<0.01
		VO_2max_	1.87 ± 0.42	1.84 ± 0.38	1.91 ± 0.40	1.92 ± 0.44	8.99	<0.01
Total		BMI Z	0.48 ± 0.74	0.58 ± 0.80	0.57 ± 0.77	0.53 ± 0.76	22.65	<0.01
		VO_2max_	2.25 ± 0.81	2.24 ± 0.79	2.40 ± 0.78	2.37 ± 0.79	78.80	<0.01

Model 1 was composed of age (continuous variable), sex (male = 1, female = 2), and BMI Z (normal weight = 1, overweight and obese = 2, weight loss = 3) and new coronavirus infection years (2019, 2020 = 1; 2021, 2022 = 2), followed by multiple linear stepwise regression analysis. Table 2 indicates that the change of cardiopulmonary endurance may be explained by the BMI Z score and the new coronavirus infection year (△*R^2^* = 0.179) after the model 2 contains BMI Z score and new coronavirus infection year (*p* < 0.01). There was a significant positive correlation between overweight and obesity in adolescents (*B* = 0.643, 95%*CI* = 0.634~0.652) and maximal oxygen absorption (*p* < 0.01). The LBW of adolescents (*B* = −0.510, 95%*CI* = −0.537~−0.484) had a negative correlation with the maximal oxygen absorption (*p* < 0.01). There was a significant positive correlation between the year of novel coronavirus infection (*B* = 0.116, 95%*CI* = 0.107~0.125) and maximal oxygen absorption (*p* < 0.01; Table 3).

**Table 3:** Multiple linear regression analysis of body mass index and maximum oxygen uptake among 12–14-year-old adolescents in Jinan City from 2019 to 2022.

Model	Constants and independent variables		standard error	*B* (95%*CI*)	t	*P*
Model 1	Constants		0.048	0.878 (0.785~0.972)	18.329	<0.01
	Age		0.004	0.242 (0.235~0.249)	66.954	<0.01
	Gender		0.006	−1.163 (−1.175~−1.152)	−196.043	<0.01
Model 2	Constants		0.036	0.403 (0.331~0.474)	11.046	<0.01
	Age		0.003	0.240 (0.234~0.245)	88.387	<0.01
	Gender		0.004	−1.090 (−1.098~−1.081)	−243.079	<0.01
	BMI Z	Overweight and obesity	0.005	0.643 (0.634~0.652)	137.942	<0.01
		Low weight	0.014	−0.510 (−0.537~−0.484)	−37.625	<0.01
	Year of infection		0.004	0.116 (0.107~0.125)	26.097	<0.01

## Discussion

This study conducted a large-scale longitudinal data collection and analysis on the body shape and cardiopulmonary health of Chinese adolescents before and after the COVID-19. The result shows that the BMI Z score of adolescents 12–14 in 2020 has increased significantly compared to 2019, and have been controlled to decrease in 2021 and 2022. The VO_2max_ for young people fell in 2020 compared to 2019 and was below the thresholds for 2019, 2021, and 2022. Moreover, the VO_2max_ of adolescents of different sexes and ages showed varying degrees of decline in 2020, and achieved rapid recovery and rebound in 2021 and 2022. These findings suggest that COVID-19 has an adverse effect on the body shape and heart and lung health of young people. Along with the increase of epidemic control and adaptation, young people show better health and heart function.

The results show that the BMI Z score of young people in the period of COVID-19 is higher, which is in line with the related findings (23–25). There is a close relationship between the young people’s physique and their life style. The related research indicates that the new coronavirus has a significant negative impact on young people’s health and promotes their life style (26). Related studies have pointed out that there have been significant changes in the lifestyle of adolescents during lockdown periods, such as: high consumption of consolatory food, less sport activities, more time spent in front of screens, sleep alteration associated with increased anxiety (27–29). These factors acted together to lead to an increase in BMI among adolescents. After the epidemic, along with the increase of the exercise time, the healthy living level has been raised, which is the basis for the improvement of the young people’s physique and health. Furthermore, the percentage of young people who consume vegetables and fruits daily has risen from 35.2% and 25.5% before the outbreak to 43% and 33.2% respectively, which has a positive impact on the improvement of physique (30).

The results indicate that the maximum oxygen uptake of young people of different ages and genders varies from 2019 to 2022, with the highest to lowest being in 2021, 2022, 2019, and 2020; The performance and lung capacity of adolescents in the 1,000/800 m race also showed varying degrees of decline in 2020, and achieved recovery and development in 2021 and 2022, with statistically significant fluctuations in lung capacity and other indicators from 2019 to 2022. Following the outbreak of new coronavirus infection, the decline of heart and lung capacity was correlated with the time and life style. According to the related studies, in the early period of novel coronavirus infection, the time of physical activity and the level of promoting health of the youth decreased significantly (31), and the decrease of aerobic activity time, diet nutrition consciousness, and health literacy also influenced the rate of heart and lung endurance (32), which decreased. Along with the recovery of physical activity, the decline tendency of heart and lung endurance was relieved, and gradually increased.

The results of multi-linear regression analysis of BMI and cardiovascular endurance showed that there was a significant positive correlation between BMI and cardiovascular endurance in adolescents, but there was a negative correlation between BMI and cardiovascular endurance. This was in line with the findings of related studies (33). According to the data collected in this study, the percentage of overweight students was higher, and their muscle mass was higher than that of thin ones, so they performed better in the 1,000 m/800 m cardiovascular endurance test. Moreover, the year of new coronavirus infection also has influence on young people’s heart and lung endurance. Along with the increase of new coronavirus infection, young people have more time to rest and exercise, and their heart and lung capacity has also been improved.

However, there are some limitations in this study. First of all, the study, which collected and analyzed physical appearance and heart and lung health data for high school students, did not take into account the whole growth cycle of teenagers. Therefore, it is necessary to take a comprehensive consideration of the developmental state of adolescents at different stages. In future research, attention should be paid to the health changes of adolescents at all ages and regions before and after the COVID-19 epidemic. Secondly, as more than 95% of the participants were from Jinan City in Shandong Province, it was not a comprehensive representation of Chinese teenagers. Studies with a larger sample size, including participants from various provinces, and other cohorts (e.g., age), are justified in reviewing and validating the observations recorded here. Moreover, in addition to body mass index and cardiopulmonary endurance, we still need to pay attention to and consider the possible impact of the COVID-19 epidemic on other health related variables of adolescents. In the future, we need to further include multivariate research to more comprehensively analyze the impact of COVID-19 epidemic on the health level and health behavior of adolescents.

## Conclusion

This study analyzed the impact of the COVID-19 on the body shape and cardiopulmonary health of Chinese adolescents. In 2020, the BMI Z score and VO_2max_ of adolescents aged 12 to 14 showed a significant rise and fall in comparison with 2019 and were controlled and developed in 2021 and 2022. There was a positive correlation between the year of new coronavirus infection and the maximal oxygen absorption.

## Data availability statement

The original contributions presented in the study are included in the article/supplementary material, further inquiries can be directed to the corresponding author.

## Ethics statement

The study protocol was approved by Shandong Normal University Ethics Committee (2021066). The studies were conducted in accordance with the local legislation and institutional requirements. Written informed consent for participation in this study was provided by the participants’ legal guardians/next of kin. Written informed consent was obtained from the individual(s), and minor(s)’ legal guardian/next of kin, for the publication of any potentially identifiable images or data included in this article.

## Author contributions

HC: Writing – original draft, Writing – review & editing. LJ: Investigation, Writing – review & editing. BL: Writing – review & editing.
